# Is it Ever Acceptable to SQUEEZE Charge Balance?

**DOI:** 10.1063/4.0001003

**Published:** 2025-10-27

**Authors:** Toby J Woods

**Affiliations:** University of Illinois at Urbana-Champaign, Urbana, IL, USA

## Abstract

When the void spaces of a structure contain highly disordered solvent molecules that cannot be adequately modeled, the SQUEEZE routine of PLATON or Solvent Mask in Olex2 are often used as a last resort to account for the electron density in the void spaces. But, what to do when it is not solvent molecules that are disordered? What if the counter-ions for a charged target molecule are so disordered that they cannot be definitively identified in the difference map? Is there a point at which there is enough additional characterization of a compound that squeezing charge balance could be considered acceptable?

